# Decoding the principle of cell-fate determination for its reverse control

**DOI:** 10.1038/s41540-024-00372-2

**Published:** 2024-05-06

**Authors:** Jonghoon Lee, Namhee Kim, Kwang-Hyun Cho

**Affiliations:** 1 Laboratory for Systems Biology and Bio-inspired Engineering, Department of Bio and Brain Engineering, Korea Advanced Institute of Science and Technology (KAIST), Daejeon, 34141 Republic of Korea; 2 Present Address: biorevert, Inc., Daejeon, Republic of Korea

**Keywords:** Systems biology, Computational biology and bioinformatics

## Abstract

Understanding and manipulating cell fate determination is pivotal in biology. Cell fate is determined by intricate and nonlinear interactions among molecules, making mathematical model-based quantitative analysis indispensable for its elucidation. Nevertheless, obtaining the essential dynamic experimental data for model development has been a significant obstacle. However, recent advancements in large-scale omics data technology are providing the necessary foundation for developing such models. Based on accumulated experimental evidence, we can postulate that cell fate is governed by a limited number of core regulatory circuits. Following this concept, we present a conceptual control framework that leverages single-cell RNA-seq data for dynamic molecular regulatory network modeling, aiming to identify and manipulate core regulatory circuits and their master regulators to drive desired cellular state transitions. We illustrate the proposed framework by applying it to the reversion of lung cancer cell states, although it is more broadly applicable to understanding and controlling a wide range of cell-fate determination processes.

## Introduction

Cell-fate determination is an evolutionarily well-conserved process through which cells make critical decisions regarding their ultimate roles within a multicellular organism^1^. This foundational process underpins functions that are indispensable for all multicellular organisms, including the precise orchestration of normal developmental pathways, the maintenance of internal equilibrium (homeostasis), and the facilitation of adult tissue regeneration. Due to its vital importance, cell-fate determination has evolved to be highly resilient to various perturbations. Nevertheless, seminal experimental findings have revealed that the predetermined destiny of a cell can be dramatically reshaped by a few molecular modifications. For instance, the overexpression of Yamanaka factors OCT4, SOX2, KLF4, and c-MYC (OSKM)-can reprogram differentiated fibroblasts into induced pluripotent stem cells^2^. Moreover, ectopic activation of Yes-associated protein can transdifferentiate terminally differentiated hepatocytes into biliary epithelial-like cells^3^. In addition, adenomatous polyposis coli restoration is able to reverse colon carcinoma cells back to a functionally normal state despite the presence of potent oncogenic mutations^4^. These findings collectively highlight that predisposed cell fates, in principle, can be changed by manipulating a few specific molecules, called master regulators, while remaining highly robust against most other molecular perturbations. This perspective further raises the following challenges: how can we identify the master regulators, and through what molecular regulatory mechanisms do they induce cell-fate changes?

Cells are dynamic systems composed of intricate signaling pathways interconnected by various feedbacks and crosstalks, forming a complex network^5,6^. The regulatory relationships within this network are predominantly nonlinear, adding layers of complexity that defy simple, intuitive predictions about how altering a specific molecule might impact cellular functions^7,8^. The inherent complexity and nonlinearity present significant challenges in understanding the principles underlying cell-fate determination and its control. To comprehend the intricate networks of intracellular molecular regulations, a systems biology approach that integrates quantitative mathematical modeling with molecular experimentation is indispensable.

In this perspective, we argue that the integration of newly emerging dynamic information with mathematical models enables us to not only decode the fundamental principles embedded in the process of cell-fate determination, but also to exert control over this intricate process to the degree that was previously unachievable. In particular, we propose that although cell-fate determination accompanies genome-wide molecular state changes, it might be underpinned by only specific subnetworks within the network, which we refer to as ‘core regulatory circuits’. These circuits are instrumental in orchestrating the intricate sequence of events that determine cell fate. Based on this, we present a conceptual framework that employs single-cell RNA-sequencing (scRNA-seq) data to identify and manipulate core regulatory circuits determining cell fate. By applying this framework to scRNA-seq data of tumorigenesis, we propose a system-level approach to identify molecular candidates for cancer reversion and their molecular mechanisms through which deregulated gene regulatory dynamics are rewired, and cancer hallmarks can be compromised to reestablish normal phenotypes.

## Representing cell-fate changes by complex molecular network dynamics

**Fig. 1:**
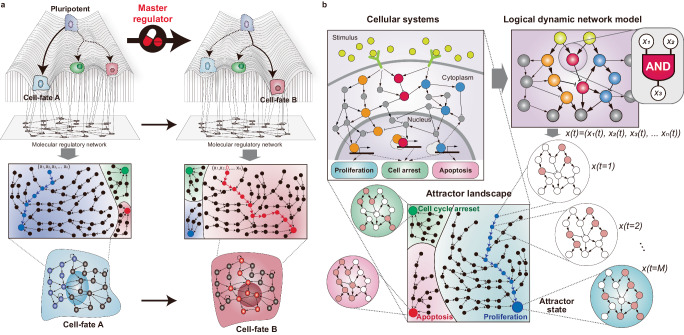
Cell-fate changes through the lens of the complex dynamics of molecular networks. **a** Cell-fate changes in the epigenetic landscape. Waddington’s landscape is widely recognized as a conceptual framework to comprehend the transitions of pluripotent cellular states toward specific valleys that represent distinct cell fates. In the landscape metaphor, these cell fates are separated by an epigenetic barrier that restricts transitions, yet the perturbation of a specific master regulator can overcome this barrier to induce a dramatic transition between cell fates (top). Each cell fate can be regarded as the most stable state (also referred to an attractor) within the attractor landscape, which represents their transition trajectories (middle). Since each cell fate is characterized by the gene activities of the underlying molecular regulatory network, altering these complex molecular interactions by regulating a specific master regulator can reshape the attractor landscape, causing it to converge into a specific cell fate (bottom). **b** A mathematical model to unravel the hidden molecular regulation logic of cellular systems. A mathematical model can describe the dynamics of cellular systems in terms of gene regulatory interactions. The model can formalize cellular phenotypes such as proliferation, cell cycle arrest, and apoptosis, by assembling molecular components into the network. For example, a logical dynamic model is composed of n genes, whose state at time *t*, defined by $x(t)=\left({x}_{1}\left(t\right),\ldots ,{x}_{n}(t)\right)$, corresponds to a point in a n-dimensional gene expression state space represented by the attractor landscape. The state of each molecule is influenced by its dynamic regulations, where the future state of each molecule can be determined by a logical equation (e.g., AND gate) of its upstream molecules. A cellular state evolves through the logical equations of all molecules in the network, eventually converging on a specific attractor at time *t* = M.

Cellular functions are orchestrated by a gene regulatory network (GRN) composed of tens of thousands of genes, intertwined through intricate nonlinear interactions. Network dynamics can be conceptualized through Waddington’s landscape, an intuitive model that illustrates how cells navigate through various states within a high-dimensional state space^9^ (Fig. 1a). In this landscape, valleys correspond to specific cell types, known as ‘attractors’, representing stable states that cells naturally settle into^10^. Building on this, the concept of ‘attractor landscape’ further illustrates the array of potentially stable states that cells can adopt. The basins surrounding these attractors indicate the probability of cells adopting each phenotype, offering insights into the dynamics and probabilities of cell-fate transitions. In this context, cell fates can be regarded as the most probable states a cell can occupy within the attractor landscape, reflecting their potential transition trajectories. In principle, the predetermined cell fates can be changed by altering the attractor landscapes of cells by rewiring the core regulatory circuits that underlie their complex molecular interactions^11,12^ (Fig. 1a). Can Waddington’s landscape metaphor be used for a quantitative description of actual cell-fate determination? Moreover, can it be applied to cell-fate inference and control?

Such a metaphorical landscape can be quantitatively described through mathematical models (Fig. 1b). These models formalize the state of a cell at time *t* by the collective value of each molecular state in the network. For example, a network composed of *n* genes can be represented at time *t* by the state vector ${x}(t)=\left({x}_{1}\left(t\right),{x}_{2}\left(t\right),\ldots ,{x}_{n}(t)\right)$. In this vector, ${x}_{i}\left(t\right)$ for $i\in \left\{\mathrm{1,2},\ldots ,n\right\}$ denotes the state of the *i*th gene at time *t*, and thus, $x(t)$ corresponds to a point in an *n*-dimensional gene expression state space. The state of each molecule is influenced by the GRN, where the future state of each molecule is determined by nonlinear interactions with its upstream molecules. Consequently, a cellular state evolves over time as a nonlinear function of all molecules in the network, $x(t+1)={F}\left({x}_{1}\left(t\right),{x}_{2}\left(t\right),\ldots ,{x}_{n}(t)\right)$, converging towards a particular state (or a set of states) in the state space, which is called the attractor state. Empirical estimation of the network structure and nonlinear function parameters from experimental data is essential for a reasonably accurate model of cell-fate determination. However, obtaining the dynamic data needed for the estimation has been experimentally challenging. Fortunately, recent advances in single-cell omics and related analytical technologies can now provide the necessary temporal data required for the construction of comprehensive mathematical models.

## Methodologies available to construct mathematical models using single-cell omics data

Single-cell sequencing technologies are advancing rapidly and are now capable of analyzing datasets, including transcriptomes, epigenomes, and proteomes from hundreds of thousands of cells. In particular, RNA sequencing has emerged as the leading technique in single-cell omics^13^. Sequencing RNA at the single-cell level allows for a detailed examination of gene transcription, providing a high-dimensional fingerprint that identifies unique cellular characteristics. Consequently, scRNA-seq has become an invaluable tool for investigating cell identity and state transitions at the level of individual cells. In this perspective, we focus on scRNA-seq. We illustrate current methodologies for utilizing scRNA-seq data in studying cell-fate determination, which encompass several critical stages as summarized below.

**Fig. 2:**
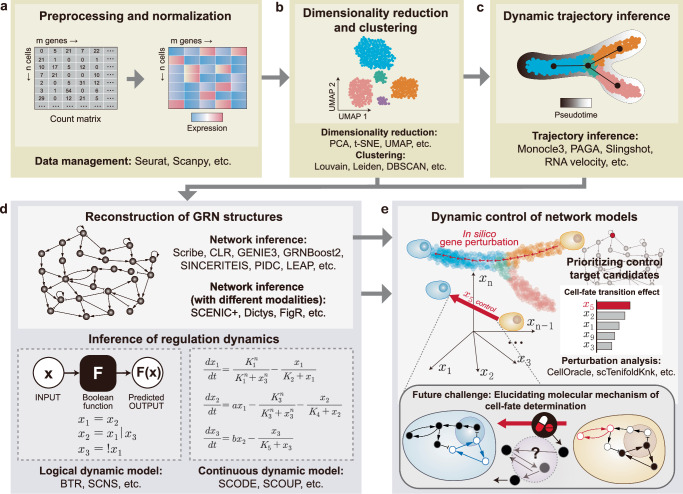
A process for constructing mathematical models using scRNA-seq data. **a** Utilization of scRNA-seq data in studying cell-fate determination. Initially, transcriptomic data is initially preprocessed to build a count matrix of cells by genes derived from raw data processing steps. Next, the count matrix is preprocessed using tools such as Seurat and Scanpy. **b** Identification of specific cell fates. Dimensionality reduction methods are applied to handle the complexity of the data, and clustering algorithms group cells with shared identities. Subsequently, specific cellular states related to the cell fates of interest are identified. **c** Inference of dynamic trajectories to reveal cell-fate changes. Cellular trajectories are derived from gene expression changes. In particular, Monocle3 and RNA velocity are generally used to infer cellular dynamic trajectories from scRNA-seq datasets. **d** Inference of GRN structures and regulation dynamics. Molecular interactions are obtained from the processed data. Furthermore, a network model can be constructed by inferring the critical regulation dynamics involved in cell-fate determination by discrete or continuous mathematical formalisms. **e** Dynamic control of network models. In silico gene perturbation analysis with tools like CellOracle and scTenifoldKnk can predict the effects of control target candidates. However, they lack insight into regulatory dynamics represented by attractor landscapes. To tackle this limitation, the future direction of research should progress beyond applying control theory to elucidate the molecular mechanisms underlying cell-fate changes.

The initial stage involves preprocessing raw count data (Fig. 2a). This crucial first step, using tools such as Seurat^14^, Scanpy^15^, and Bioconductor-based SingleCellExperiment^16^, involves rigorous quality control, normalization, and feature selection, to establish a robust foundation for subsequent analyses. Following preprocessing, the complexity of data is dealt with through dimensionality reduction techniques like PCA^17^, t-SNE^18^, and UMAP^19^, effectively simplifying the data while preserving its essential characteristics (Fig. 2b). Following dimensionality reduction, clustering algorithms such as Louvain^20^, Leiden^20^, DBSCAN^21^, and SINCERA^22^ are used to group cells with shared identities. This step is followed by the annotation of each clustered cell type using established biological knowledge, and the identification of specific cell states that correlate with the cell fates of interest.

To re-order cellular trajectories according to gene expression changes, pseudotime analysis tools like Monocle3^23^ are employed to map cell trajectories from scRNA-seq snapshots (Fig. 2c). RNA velocity^24,25^, based on vectors derived from mRNA splicing dynamics, also indicates the direction and likelihood of cell state transitions. Additional methods like Slingshot^26^, PAGA^27^, and FateID^28^ are also useful for inferring cellular dynamic trajectories and quantifying cell-fate probabilities.

The next phase involves constructing a molecular regulatory network (Fig. 2d, top), which infers molecular interactions from previously processed data^29^, often utilizing mutual information to understand nonlinear relationships within transcriptomic data. Tools such as Scribe^30^ and CLR^31^, utilize mutual information to gauge nonlinear transcriptomic relationships, and are instrumental in building the molecular regulatory network. GENIE3^32^, GRNBoost2^33^, PIDC^34^, LEAP^35^, and SCENIC^36^ are also widely used for this purpose. Furthermore, by integrating different single-cell data modalities (e.g., gene expression, chromatin accessibility), SCENIC+^37^, Pando^38^, Dictys^39^, and CellOracle^40^ can estimate the regulatory effect of each transcription factor (TF) on each gene mediated by specific regions of DNA, and then infer the more specific GRN structure.

In addition to the constructed GRN structure, mathematical models are constructed by inferring and encapsulating critical regulatory dynamics from scRNA-seq data (Fig. 2d, bottom). To construct a logical dynamic model, methods such as BTR (BoolTraineR)^41^ and SCNS (single cell network synthesis)^42^ can be employed. Building a Boolean network model requires optimizing Boolean regulation logic. This process is tailored to each algorithm used, such as the Z3 solver and the Quine-McCluskey (QM) algorithm, and is performed on binarized data to ensure optimal precision. Parallel to this, continuous dynamic modeling adopts a different approach. This method often involves deriving ordinary differential equations (ODEs) or stochastic differential equations, with a particular emphasis on pseudotime ordering of the data^43–45^. For example, SCODE^44^ employs linear regression techniques, whereas SCOUP^45^ utilizes a continuous diffusion process and is designed to analyze single-cell expression data during differentiation processes. This latter model, based on the Ornstein–Uhlenbeck process, is especially adept at determining the interdependencies between gene expression levels at distinct temporal points. This method offers a more detailed understanding of cellular dynamics across various time points.

To summarize, we provide a brief overview of useful methods for constructing mathematical models (Table 1). These models are invaluable for identifying molecular targets, particularly master regulators, through extensive systematic perturbation analysis using tools like CellOracle^40^ and scTenifoldKnk^46^, which predict gene perturbation effects and identify key cell-fate regulators (Fig. 2e). However, despite their advanced capabilities, these methods require laborious processes that involve individually controlling molecules and analyzing complex systems. Furthermore, these tools do not inherently incorporate an understanding of system dynamics, such as the attractor landscape, into their analyses. This poses significant limitations on revealing the specific molecular regulatory mechanisms that dictate cell-fate decisions. To address these challenges, control theory may offer a new path forward.

**Table 1:** A brief overview of available methodologies to construct network models

Methods	Possible input data types		Type of network interactions		Analysis of regulation dynamics	Default motif database	Implementation	URL
	scRNA -seq	scATAC -seq	Signed	Weighted				
GENIE3^32^/ GRNBoost2^33^	O	X	X	O	X	X	Python and R	https://github.com/aertslab
SINCERETIES^79^	O	X	O	X	X	X	R and MATLAB	https://github.com/CABSEL/SINCERITIES
PIDC^34^	O	X	X	X	X	X	Julia	https://github.com/Tchanders/NetworkInference.jl
LEAP^35^	O	X	O	O	X	X	R	R package LEAP available on CRAN
SCENIC^36^	O	X	O	O	X	cisTarget	Python and R	https://scenic.aertslab.org/
SCENIC+^37^	O	O	O	O	X	cisTarget	Python and R	https://github.com/aertslab/scenicplus
scMTNI^80^	O	O	X	O	X	CIS-BP	C++	https://github.com/Roy-lab/scMTNI
Pando^38^	O	O	O	X	X	CIS-BP	R	https://github.com/quadbio/Pando
CellOracle^40^	O	O	O	O	X	CIS-BP	Python	https://github.com/morris-lab/CellOracle
FigR^81^	O	O	O	X	X	CIS-BP	R	https://github.com/buenrostrolab/FigR
Dictys^39^	O	O	O	O	X	HOCOMOCO	Python	https://github.com/pinellolab/dictys
scTenifoldKnk^46^	O	X	O	O	X	X	R and MATLAB	https://github.com/cailab-tamu/scTenifoldKnk
BTR^41^	O	X	O	X	O	X	R	https://github.com/cheeyeelim/btr
SCNS^42^	O	X	O	X	O	X	F# and Javascript	https://github.com/swoodhouse/SCNS-GUI
SCODE^44^	O	X	O	O	O	X	R and Julia	https://github.com/hmatsu1226/SCODE
SCOUP^45^	O	X	O	X	O	X	C++	https://github.com/hmatsu1226/SCOUP

## Emerging significance of applying control theory to explore core regulatory circuits and their master regulators

Control theory is a field of study that focuses on system characterization and manipulation. It involves understanding how systems behave and devising methods to achieve desired outcomes through control actions. This discipline has evolved over time to address vital challenges in complex systems. In the early 20th century, Black’s development of the negative feedback amplifier laid the groundwork for feedback control of simple systems^47^. In the 21st century, Wolkenhauer, Kitano, and Cho introduced the interdisciplinary principles of systems biology, merging control theory with high-throughput technologies for cellular research^48^. Aligned with this evolution, in 2011, Barabási and Slotine made a significant advancement in complex network control by integrating network science with control theory^49^. Their works highlighted the future focus on addressing the intricate interplay of elements in a nonlinear, networked system.

Current developments in complex network control theories have evolved in two principal directions: one that focuses on the structural characteristics of networks and another that considers the inherent nonlinear dynamics within these networks. Controlling network centrality determines the most influential regulators in network interactions using metrics such as degree centrality, betweenness centrality, and eigenvector centrality^50^. This approach often aims to identify a minimal subset of driver nodes required to direct a network from any given state to a specific desired state^51^. In contrast, strategies for controlling network dynamics, like logical domain of influence (LDOI)^52^, feedback vertex set (FVS)^53^, stable motif^54^, searching for differential expressed positive circuits (DEPCs)^55^, and global stabilization analysis^56^, were suggested to manage state transitions caused by the network dynamics. These methods collectively aim not only to identify ‘control targets’, defined as a specific set of nodes capable of steering the system towards a set of desired states, but also offer tools for analyzing molecular regulatory mechanisms. For instance, stable motif analysis has been instrumental in uncovering crucial feedback loops that govern processes like the epithelial-to-mesenchymal transition^57^, leukemia cell-fate decisions^54^, and the differentiation of helper T cells^54^. In addition, the LDOI approach has identified key control targets and their influences, and the DEPC method has discovered related positive circuits to stabilize a certain attractor^55^. While these methods provide solutions for controlling theoretical model systems, some result in suboptimal solutions with unnecessary targets, and yet others face scalability issues, rendering them limited to very small-scale models. Furthermore, to our knowledge, there has been no attempt to systematically identify both control targets and the resulting fate-determining paths (or circuits) revealing specific molecular regulatory mechanisms. Thus, identifying optimal control targets (i.e., master regulators) and the resulting core regulatory circuits for real-world cell-fate control remains a significant challenge.

**Fig. 3:**
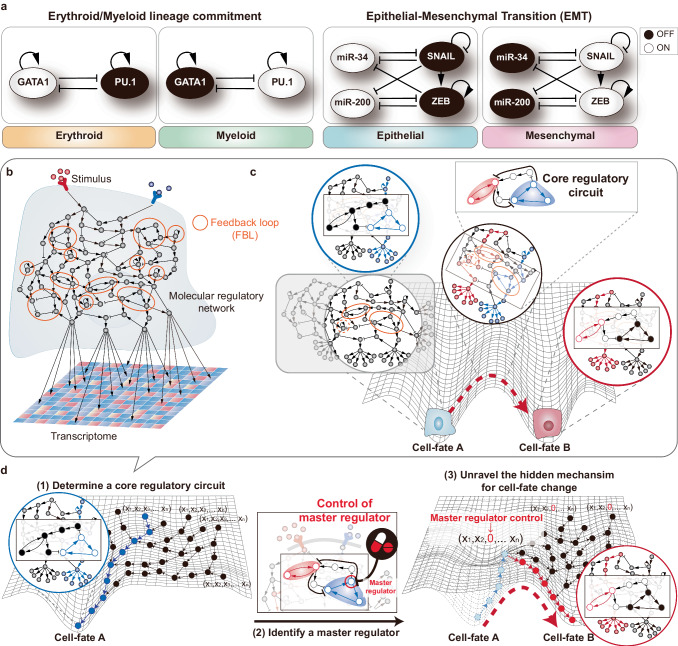
Core regulatory circuits in a dynamic network model. **a** Examples of key regulatory components for cell-fate determination. **b** Representation of regulation dynamics at the network-level. The molecular regulatory network is useful to describe the regulation dynamics inferred from transcriptome data in response to a stimulus. From these molecular regulations, all feedback loops are investigated to find out an essential subnetwork. **c** Core regulatory circuit for cell-fate determination. As each cell fate is defined by the gene activities within the molecular regulatory network, alteration in a few critical molecular interactions can lead to a shift towards a specific cell fate. In other words, a specific subnetwork composed of interconnected positive feedback loops (referred to as a ‘core regulatory circuit’) is considered the primary driver of cell-fate changes. For example, it could be a toggle switch, a well-known designed circuit in cell-fate decision-making. **d** Elucidating the molecular mechanism through a core regulatory circuit and its master regulator. Each cell fate can be regarded as an attractor within the attractor landscape that includes the transition trajectory. First, based on detailed analysis as outlined in **b**, **c**, a core regulatory circuit essential for cell-fate determination can be extracted. Next, a master regulator that governs the change of cellular states described by the core regulatory circuit can be identified. Finally, the hidden mechanism underlying the transition when the master regulator is controlled can be unraveled.

To address the aforementioned challenge, leveraging the key biological features that play a role in cell-fate determination could be an essential strategy. Many previous experimental studies have already shown that while cell systems are comprised of a complex large network, a core regulatory circuit, composed of only a few key molecules, plays a crucial role in determining cell fates^58–61^. In particular, such core regulatory circuits are often composed of multiple nested feedback loops, with at least one being a positive feedback loop. For example, in quorum sensing, molecules like N-acyl homoserine lactone (AHL) in bacteria facilitate population-wide communication^62^; the regulatory feedback between TFs OCT4, SOX2, and NANOG maintains pluripotency in embryonic stem cells^63^; the double negative feedback loop between PU.1 and GATA1 controls the erythroid versus myeloid lineage commitment^64^; and the interconnected SNAIL/miR-34 and ZEB/miR-200 feedback loops are key regulatory components of the epithelial–mesenchymal transition (EMT) process in cancer metastasis^65^ (Fig. 3a). Furthermore, combinations of feedback and feedforward controls can generate biological transitions reliably in noisy environments where the activity of individual components can vary over a range of parameters^66^. Those previous studies indicate the significance of specific feedback mechanisms within core regulatory circuits in governing crucial cellular processes. This leads to the following question: how can we identify these core regulatory circuits and their master regulators?

To computationally resolve this problem, the concept of a network kernel has been introduced, encompassing the ‘kernel’ method for simplifying networks^67^ and the ‘control kernel’ approach for altering attractor landscapes with minimal regulators^68,69^. These kernel-based approaches are crucial for focusing on and controlling the core regulatory circuits that govern vital biological phenomena. Similarly, since cellular circuits with positive feedback loops induce multistationary behavior, identifying and analyzing the properties of feedback loops can offer critical clues to determine core regulatory circuits within large networks^69–71^. Another algorithm, ‘OpitCon’, can identify combination targets using a subgraph based on structural controllability theory, and then describe specific core downstream subnetworks and their crosstalk links that contribute to therapy resistance^72^. More recently, Rukhlenko et al.^73^ developed a novel framework called ‘cSTAR’. This method uses omics data to classify cell states and transforms them into mechanistic models. These models consist of a key molecular regulatory network that is instrumental in controlling the attractor landscape that governs cell-fate determination. Moreover, systematic perturbation to the model helps to identify control targets for the desired change in cell fate.

Based on previous studies, we postulate that core regulatory circuits consist of subnetworks interconnected by feedback loops, and that these circuits are the primary drivers of cell fate. Further advancing this concept, we can propose a detailed procedure as follows. First, we can investigate all feedback loops associated with different cellular states of interest (Fig. 3b). Subsequently, through an analysis of network dynamics influenced by feedback loop states, we can prioritize the positive feedback loops that are instrumental in controlling the attractor landscape governing cell-fate determination (Fig. 3c). For this, we can employ previous control methods such as LDOI^52^, stable motif^54^, and DEPC^55^, or improve these methods for further exploration of key subnetworks. As a result, the prioritized feedback loops can be refined to form a core regulatory circuit. Lastly, through systematic perturbation to the model (or core regulatory circuit) or by employing the improved kernel-based methods, we can identify (minimal) master regulators for the desired cell-fate change (Fig. 3d). Together, this control theory-based approach would be a highly promising way to understand and manipulate cell-fate processes, centered around core regulatory circuits, of a dynamic system.

**Fig. 4:**
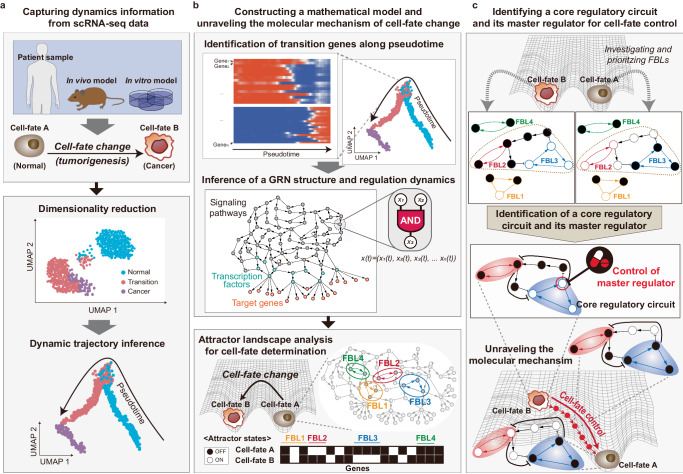
A framework for identifying core regulatory circuits and master regulators for cell-fate change. **a** Capturing critical information on dynamics from scRNA-seq data. ScRNA-seq data can be obtained from cancer samples, in vivo models, or in vitro models. From this collected dataset, the dynamics of cellular states during cell-fate changes (e.g., tumorigenesis) can be captured by the following steps (top). First, dimensionality reduction and clustering methods are generally employed to clarify normal, transition, and tumor states. Then we utilize dynamic trajectory inference methods to reveal the trajectories during cell-fate change (bottom). **b** Constructing a mathematical model to analyze the molecular mechanism. We identify transition genes that play a significant role in changing cellular states during cell-fate determination. A GRN structure and regulation dynamics are inferred by integrating these identified genes and the molecular dynamic interactions that connect them (top). The model is further investigated through attractor landscape analysis to elucidate the underlying mechanism for cell-fate determination. Based on the corresponding attractors, feedback loops can be selected from the GRN structure (bottom). **c** Identifying a core regulatory circuit and its master regulator for cell-fate control. Through evaluating and prioritizing feedback loops, we can identify a crucial network of interconnected feedback loops as a core regulatory circuit, pinpointing the most influential regulator as the master regulator. Analyzing how the states of feedback loops within this circuit change when the master regulator is controlled allows us to reveal the molecular mechanisms underlying cell-fate control.

From this, we integrate aforementioned progresses and suggest a comprehensive framework for systematically identifying core regulatory circuits and their master regulators for cell-fate change (Fig. 4). This framework consists of three interconnected components: capturing dynamic information from single-cell omics data, constructing and analyzing a mathematical model of molecular mechanisms in cell-fate determination, and identifying the candidates of master regulators for a desired cell-fate control (Fig. 4a–c). To explicitly introduce our framework, we provide an illustrative example as follows.

## Illustrative example: identifying control targets for cancer reversion by using single-cell RNA-sequencing data from lung cancer samples

Traditional anticancer therapies have focused on removing cancer cells, but their effectiveness is limited due to the inevitable emergence of resistance, which arises as a consequence of cancer cell plasticity. Cancer plasticity is an emerging hallmark of cancer cells, and it plays a crucial role in cancer initiation and progression, as well as adaptation to therapy and intra-tumoral heterogeneity^74–76^. Hence, recent research has shifted focus towards targeting highly plastic cancer cells emerging during tumorigenesis, aiming to inhibit their development.

**Fig. 5:**
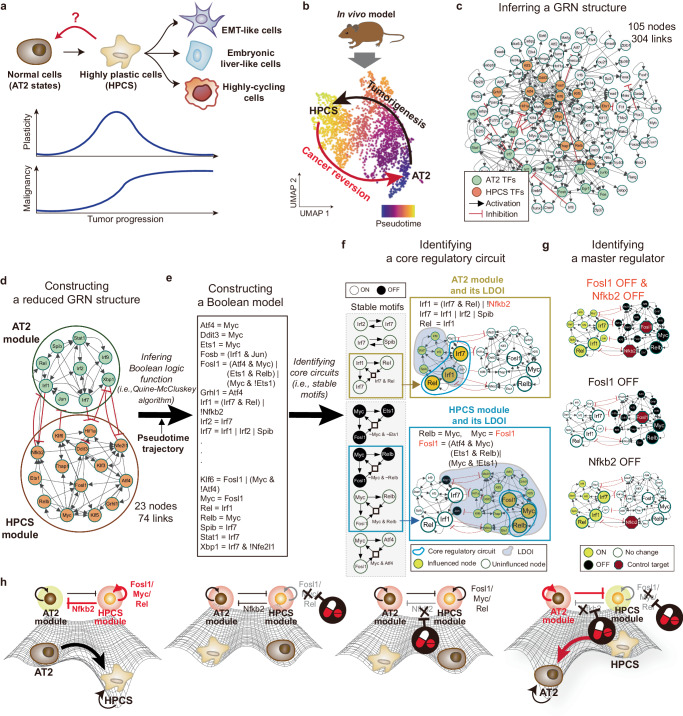
Identifying control targets for reversing lung cancer cell state. **a** Emerging role of cancer plasticity in tumorigenesis. When normal cells (e.g., AT2 states) are exposed to KRAS mutations alone or combined with TP53 mutations, these cells evolve into highly plastic cells (e.g., HPCS). This progression results in increased plasticity, leading to diversification into various cancer cell types, such as EMT-like, embryonic liver-like, and highly-cycling cells, thereby enhancing malignancy. **b** Low dimensional representation of tumorigenesis and cancer reversion. After collecting and preprocessing the dataset obtained from the in vivo model, dimensionality reduction and clustering are employed to annotate cellular identities at different stages during tumorigenesis. UMAP representation visualizes the two cellular states, AT2 and HPCS, colored by pseudotime. **c** The resulting GRN structure. A gene regulatory network is composed of critical transcription factors (TFs) that regulate gene expression changes along the lung tumorigenesis trajectory. **d** Identifying differential modules between AT2 and HPCS states. The network comprises selected feedback loops with differential activation between AT2 and HPCS. It reveals mutually activating interactions within each state and inhibitory interactions between distinct states. The structure is reminiscent of a toggle switch, a well-known design principle in cell-fate determination, suggesting a fundamental regulatory structure in tumorigenesis. **e** Inferring Boolean network model. A Boolean network model is inferred from the network structure and pseudotime trajectory by applying Quine-McCluskey algorithm. **f** Identifying a core regulatory circuit. By analyzing the Boolean network model, we can identify crucial feedback loops essential for phenotype stabilization. Assessing the impact of their LDOIs on AT2 and HPCS modules reveals a key circuit critical for phenotype stabilization. This core regulatory circuit, including Rel, Irf1, Irf7, Fosl1, Myc, and Relb, can play a crucial role in controlling cell fates. **g** Identifying a master regulator. Inhibition of only Fosl1 decreases TF activities of HPCS (top). Nfkb2 acts as a major negative regulator of the positive feedback loop among AT2 TFs (middle). Disrupting only Nfkb2 leads to the re-emergence of the AT2 states. The combined inhibition of Fosl1 and Nfkb2 effectively destabilizes HPCS, thereby facilitating a stable transition from a cancer state back to a normal AT2 state (bottom). **h** Conceptual illustration of the reversion dynamic landscape through combinatorial inhibition strategy. Targeting either molecule alone proves inadequate due to their integral feedback mechanisms. Conversely, simultaneous inhibition of both Fosl1 and Nfkb2 is necessary and effective for a significant cellular landscape shift, driving the transition toward the AT2 phenotype.

This illustrative example shows the emerging concept of ‘cancer reversion’, which seeks to transform highly plastic lung cancer cells back into normal cells, instead of merely eliminating cancer cells. Recent studies have indicated that cells with high plasticity emerge during mouse lung tumorigenesis upon introduction of KRAS mutations into AT2 cells, which are typically considered the origin of lung cancer. These cells lose their original characteristics and transition through AT1/AT2-like states, eventually diversifying into various cancer cell types such as EMT-type, embryonic liver-like, GI-like, and high-cycling cells, thereby increasing tumor heterogeneity (Fig. 5a).

In this illustrative example, we begin with obtaining time-series scRNA-seq data collected during lung tumorigenesis from AT2 cell state to high-plasticity cell state (HPCS) from a public repository^77^. This dataset provides single-cell transcriptomic information for ~2200 cells. The proposed framework entails preprocessing, dimensionality reduction, and clustering to annotate cell identities at different stages during tumorigenesis. Then, it conducts pseudotime ordering to delineate the trajectory from AT2 cells, through intermediate states, to HPCS cells, providing a comprehensive map of cellular evolution during lung tumorigenesis (Fig. 5b).

The proposed framework identifies critical TFs that can regulate gene expression changes along the lung tumorigenesis trajectory by comparing the activities of TFs among the distinct cell clusters. Then, the interactions among these TFs can be inferred, resulting in a GRN structure consisting of 105 nodes and 304 links (Fig. 5c). Next, to explore essential regulatory mechanisms associated with specific phenotypes, the framework can investigate all positive feedback loops showing differential activation between AT2 and HPCS states by evaluating their TF activities. By aggregating those feedback loops, the framework generates a reduced GRN, consisting of 23 nodes and 74 links, which contains key dynamical information (Fig. 5d). Intriguingly, TFs within the same state (e.g., AT2 or HPCS) are primarily interconnected through mutually activating positive feedback, whereas TFs across distinct states (e.g., AT2 versus HPCS) engage predominantly through mutually inhibitory interactions.

With the GRN structure and trajectory information, a Boolean logic model can be constructed to simulate the tumorigenesis process. This process involves discretizing the continuous expression values of each TF along the pseudotime trajectory (employing clustering methods like k-means). Subsequently, the influence of each TF in the GRN structure can be determined through Boolean logic functions, utilizing the QM algorithm (Fig. 5e). Using the Boolean network model, the framework can pinpoint the feedback loops of the most dynamic significance (in this example, stable motifs). These feedback loops and their corresponding LDOIs have the potential to stabilize either phenotype when modulated. Then the framework can assess the influence of the LDOIs on AT2 and HPCS modules, and prioritize key feedback loops based on their relevance to either phenotype (Fig. 5f). The most influential feedback loops can form a core regulatory circuit, consisting of Rel, Irf1, Irf7, Fosl1, Myc, and Relb. Once the core regulatory circuit is stabilized, it can fix the values of most TFs within the respective AT2 and HPCS modules.

The proposed framework then leverages information on core regulatory circuits to find control targets that can inhibit the HPCS module and activate the AT2 module. The LDOIs for individual nodes and pairs of nodes are calculated, then each LDOI is checked whether it can fix the influential feedback loops in the desired state to identify control targets. For example, a combination of Fosl1 (or Myc) and Nfkb2 can be identified as a master regulator, thereby ensuring the stable activation of the AT2 module and the deactivation of the HPCS module (Fig. 5g). Inhibition of Fosl1 can deactivate HPCS TF activities while having no impact on AT2 TFs. On the other hand, disruption of Nfkb2 can de-repress AT2 TF activities in the HPCS state, activating downstream regulators and leading to the re-emergence of the AT2 state. Combined inhibition of E2f4 and Nfkb2 can destabilize the HPCS state, and enable a stable activation of the AT2 module. This combinatorial inhibition strategy can, therefore, effectively disrupt the positive feedback within the HPCS module while also neutralizing its negative impact on the AT2 module. The result shows a stable shift from a highly plastic cancerous state back to a normal-like AT2 state. Moreover, the role of key molecules in plastic cancer cells can be illustrated using the landscape concept (Fig. 5h). In these cells, Fosl1 and Nfkb2 are highly active, stabilizing the HPCS while repressing the normal AT2 module. This ensures the persistence of the HPCS. However, to revert HPCS back to normal, merely inhibiting either Fosl1 or Nfkb2 is insufficient, as each alone does not fully shift the landscape towards the AT2 phenotype. The positive feedback of Fosl1 maintains the HPCS state, or the remaining activity of Nfkb2 suppresses the AT2 module. Only the simultaneous inhibition of both Fosl1 and Nfkb2 can significantly alter the landscape, favoring a shift towards the AT2 phenotype.

In summary, this example illustrates how systems biology can be applied to understand and manipulate cell-fate transitions in cancer. By integrating mathematical modeling with experimental data, we can identify crucial regulatory mechanisms that reverse cell states, offering new insights and potential therapeutic strategies in cancer treatment. Although this example illustrates the whole process proposed for inducing cancer reversion from public single-cell data, limitations are noted. Cell-fate determination is a complex phenomenon that encompasses changes not just at the transcriptomic level but also across various molecular levels. The continuous development of technologies that utilize single-cell multiomics data, which integrate transcriptomic, proteomic, and epigenetic data, has been pivotal. The use of such data can significantly enhance the proposed framework. In particular, the recent increase in single-cell multiomics data, combining scRNA-seq and scATAC-seq, has been noteworthy. scATAC-seq allows for the pruning of indirect regulation information from functional regulatory relationships obtained from transcriptomic data by providing information on the open chromatin regions at promoter sites. This enables the construction of more accurate GRN structures and, consequently, the development of more precise mathematical models. Furthermore, factors like the physiological state of a cell, not directly included in omics data, can significantly influence cell fate^78^. Although our framework does not directly incorporate such state information, it can be indirectly reflected through TF activity within the GRN structure and its mathematical model. The future availability of technologies for simultaneous measurement of the physiological and molecular states of a cell promises the development of models that integrate these dimensions for a comprehensive understanding.

## Conclusions

Among the various molecules within a cell, only a few key molecules have a significant impact on cell fate. What distinguishes these master regulators from other molecules? How can we identify those master regulators, and through what molecular regulatory mechanisms do they induce cell-fate changes? To answer these questions, it is necessary to analyze and understand the behavior of a huge molecular regulatory network within a cell. In the past, such attempts were limited due to experimental constraints. Yet, recent advances in single-cell omics technologies, along with a decade of progress in network control technologies, enable us to answer these fundamental questions and usher in a renaissance for systems biology. The conceptual framework we introduce can offer an unprecedented opportunity for cell fate control by integrating the latest technological innovations into a comprehensive, novel strategy.
